# Editorial: Reviews in gastroenterology 2025

**DOI:** 10.3389/fmed.2026.1867897

**Published:** 2026-05-25

**Authors:** Meijia Wei, Jisen Xu, Huan Tong

**Affiliations:** 1 West China School of Medicine, Sichuan University, Chengdu, China; 2 Department of Gastroenterology and Hepatology, West China Hospital, Sichuan University, Chengdu, China

**Keywords:** abdominal cocoon syndrome, colorectal cancer, functional gastrointestinal disorders, *Helicobacter pylori*, metabolic dysfunction-associated steatotic liver disease, pancreatic infection, postoperative nausea and vomiting

The high prevalence of gastrointestinal diseases worldwide has led to substantial disturbances in human health with increasing disease burden. The knowledge of gastrointestinal disease is expanding, and the Research Topic *Reviews in Gastroenterology 2025* includes papers involving colorectal surgery, irritable bowel syndrome, colorectal tumors, postoperative nausea and vomiting after laparoscopy, metabolic dysfunction-associated steatotic liver disease, dyspepsia, abdominal cocoon syndrome, *Helicobacter pylori*, and necrotizing pancreatitis.

Bile acids are a family of metabolites with complex functions, including regulating lipid and glucose metabolism and affecting hepatocyte injury and liver fibrosis (1). Ursodeoxycholic acid (UDCA) is metabolized from chenodeoxycholic acid by the intestinal microbiota. UDCA is widely used to treat primary biliary cholangitis; however, its efficacy in metabolic dysfunction-associated steatotic liver disease (MASLD) is still unclear. A systematic review by Khurmatullina et al. suggested that UDCA could significantly reduce the levels of hepatocyte injury biomarkers (ALT and AST) and cholestatic markers (GGT and ALP), indicating that UDCA has liver-protective and cholestasis-modulating effects. Therefore, UDCA could become a potential option for the management of MASLD, especially for metabolic dysfunction-associated steatotic hepatitis.

Abdominal cocoon syndrome (ACS) is a rare but challenging disease. Chen et al. summarized the etiology, pathogenesis, clinical manifestations, diagnosis, and treatment of this disease. Tuberculosis, autoimmune response, repeated abdominal membrane irritation, and peritoneal dialysis can contribute to ACS, which is characterized by the encapsulation of the small intestine by dense fibrous membranes obstructing the intestine. It is often misdiagnosed, although enhanced CT is an important diagnostic method. Mild intestinal obstruction can be treated conservatively, whereas intestinal obstruction, intestinal ischemia, perforation, and surgical intervention are needed. This review also highlights the molecular and cellular mechanisms of ACS, offering deeper insight into this disease.

Inflammatory bowel disease (IBD) is a chronic inflammatory disorder of the gastrointestinal tract involving host–microbiota interactions. Although the prevalence of *Helicobacter pylori* infection is low in the IBD population, large sample size studies are needed to validate the conclusions. Bi reviewed 44 case–control studies with a total sample size of 305,452 (Bi et al.). *Helicobacter pylori* infection is inversely associated with IBD, and this negative relationship is more prominent in Crohn's disease, Eastern, and younger (age ≤ 40 years) populations. *Helicobacter pylori* seems to be a protective factor against IBD. Although the underlying mechanism is yet to be properly understood, this phenomenon should alert physicians to thoroughly evaluate the benefit and risk of *H. pylori* eradiation in IBD patients during decision-making.

Approximately 20% of acute pancreatitis cases progress to moderately severe pancreatitis, of which 20% develop infection to make the clinical situation life-threatening, with the mortality at least double (2). Therefore, prevention of pancreatic infection is crucial for improving the clinical outcome of acute pancreatitis. The review authored by Jaknov et al. lists variables indicating pancreatic infection of acute pancreatitis, including C-reactive protein, blood urea nitrogen, urea concentration, procalcitonin, interleukin 6 and interleukin 8. This review highlights plausible modalities for preventing pancreatic infection, the strength of which are ranked in descending order as early enteral nutrition, selective digestive tract decontamination, prophylactic antibiotics, and probiotics (Jaknov et al.). It provides a priority list of prevention measures for pancreatic patients with acute pancreatitis.

The efficacy of vitamin D in gastrointestinal disease has become a hot topic in recent decades. A preclinical study revealed that vitamin D promotes the proliferation of intestinal microbiota through immune cells, thereby regulating immunity against cancer (3). Whether the preclinical findings work well in the human body is answered by the review authored by Naji et al. Vitamin D has a protective effect on the development of colorectal cancer in humans, but the evidence of the effect of vitamin D supplementation on the prognosis of patients with colorectal cancer is insufficient. Obesity and genetic factors might affect the effect of vitamin D on the prognosis of patients with colorectal cancer. Hence, the conclusions on this topic are not completely clear and still demand further study.

In addition to the review on vitamin D and colorectal cancer, there are two additional reviews on colorectal cancer. One review focused on postoperative surgical site infections (SSI). SSI increases extra suffering and cost to patients. Oral antibiotic bowel preparation for decreasing SSI was evaluated in this review (Lu et al.). Compared with MBP alone, oral antibiotic bowel preparation (OABP) combined with mechanical bowel preparation (MBP) significantly reduces both the incidence of SSI and anastomotic leakage. OABP is effective at reducing not only superficial SSI but also deep SSI and organ and space infections. However, the name, dosage and duration are not presented in the review. Another review compared the pocket-creation method (PCM-ESD) with conventional ESD (C-ESD) in terms of procedural efficiency and safety for treating colorectal cancer (Jiekang et al.). These findings suggest that compared with non-granular-type tumors, non-granular-type tumors are associated with higher en bloc resection rates and R0 resection rates, with advantages for fibrotic lesions and laterally spreading tumors, as well as a lower perforation risk. However, these pooled estimates derive predominantly from cohort studies rather than the two included randomized controlled trials, raising the possibility of selection bias and learning-curve effects inflating apparent benefits. In addition, it remains unclear whether procedural advantages translate into improved survival or sustained symptom relief as quality-of-life metrics. Long-term oncologic outcomes were not assessed.

Postoperative nausea and vomiting (PONV) remains a common complication after laparoscopic surgery because of pneumoperitoneum-induced vagal stimulation, and routine antiemetic prophylaxis often provides only partial relief. Qin et al. 12 conducted a meta-analysis of 9 randomized controlled trials to evaluate whether transcutaneous electrical acupoint stimulation (TEAS) could serve as an effective adjunctive therapy. This meta-analysis revealed that TEAS significantly reduces the incidence of PONV. TEAS shortens the time to first flatus in nongynecological surgeries rather than in gynecological surgeries. TEAS seems to be an effective alternative treatment for PONV after laparoscopic surgery. However, TEAS parameters such as acupoints, frequency, intensity, and duration require standardization.

Functional gastrointestinal disorders such as functional dyspepsia (FD) and irritable bowel syndrome (IBS) are prevalent among different ethnicities. Not only developed countries but also developing countries have a high prevalence of IBS. Vo and Luu integrated 21 Vietnamese studies on IBS, reporting a wide prevalence ranging from 5.5% to 68.2%, along with frequent psychological comorbidities and preliminary benefits from probiotics and counseling. However, the studies included in the review differ in terms of study population, and the actual disease burden is unclear because of study heterogeneity.

In terms of other functional gastrointestinal disorders, FD is a relapsing disorder that may coexist with anxiety and depression. In addition to medicine, acupuncture has become another treatment, but it is controversial. A meta-analysis of 23 randomized controlled trials revealed that acupuncture improves FD symptoms and quality of life without increasing adverse events, although evidence for psychological outcomes remains insufficient (Li et al.). In addition, blinding remains challenging in acupuncture trials, and short-term follow-up is insufficient for the evaluation of FD treatment; acupuncture protocols varied across the included studies, affecting the evaluation accuracy of acupuncture treatment in FD patients.

*Reviews in Gastroenterology 2025* offers new insights into many aspects of gastroenterology, and these topics on gastroenterology are not conclusive, as new knowledge is emerging and accumulating constantly. *Reviews in Gastroenterology 2026* is also expected to share more opinions on clinical issues related to gastroenterology.
